# Artificial neural networks for non-linear age correction of diffusion metrics in the brain

**DOI:** 10.3389/fnagi.2022.999787

**Published:** 2022-10-20

**Authors:** Thomas D. Kocar, Anna Behler, Christoph Leinert, Michael Denkinger, Albert C. Ludolph, Hans-Peter Müller, Jan Kassubek

**Affiliations:** ^1^Department of Neurology, University of Ulm, Ulm, Germany; ^2^Geriatric Center Ulm, Agaplesion Bethesda Ulm, University of Ulm, Ulm, Germany; ^3^Institute of Geriatric Research, Ulm University Medical Center, Ulm, Germany; ^4^German Center for Neurodegenerative Diseases (DZNE), Ulm, Germany

**Keywords:** diffusion tensor imaging, age dependence, magnetic resonance imaging, fractional anisotropy, diffusivity, machine learning, neural network

## Abstract

Human aging is characterized by progressive loss of physiological functions. To assess changes in the brain that occur with increasing age, the concept of brain aging has gained momentum in neuroimaging with recent advancements in statistical regression and machine learning (ML). A common technique to assess the brain age of a person is, first, fitting a regression model to neuroimaging data from a group of healthy subjects, and then, using the resulting model for age prediction. Although multiparametric MRI-based models generally perform best, models solely based on diffusion tensor imaging have achieved similar results, with the benefits of faster data acquisition and better replicability across scanners and field strengths. In the present study, we developed an artificial neural network (ANN) for brain age prediction based upon tract-based fractional anisotropy (FA). Consequently, we investigated if this age-prediction model could also be used for non-linear age correction of white matter diffusion metrics in healthy adults. The brain age prediction accuracy of the ANN (*R*^2^ = 0.47) was similar to established multimodal models. The comparison of the ANN-based age-corrected FA with the tract-wise linear age-corrected FA resulted in an *R*^2^ value of 0.90 [0.82; 0.93] and a mean difference of 0.00 [−0.04; 0.05] for all tract systems combined. In conclusion, this study demonstrated the applicability of complex ANN models to non-linear age correction of tract-based diffusion metrics as a proof of concept.

## Introduction

Human aging is characterized by progressive loss of physiological functions (López-Otín et al., 2013). In the brain, aging specifically affects the frontal lobe and, in contrast, relatively spares posterior areas and infratentorial pathways (Raz and Rodrigue, 2006; Cox et al., 2016; Behler et al., 2021). Recently, the concept of brain aging has gained momentum (Baecker et al., 2021), as advanced statistical regression and machine learning (ML) models have opened new possibilities in analyzing neuroimaging data. A common approach to assess the so-called brain age of a person is fitting a regression model to neuroimaging data from a group of healthy subjects with their chronological age as the target variable and then using the resulting model for individual age prediction (Cole and Franke, 2017). This prediction, rather than chronological age, is considered to reflect a person’s brain age which may, to a certain extent, reflect brain “health.” The difference between the chronological age and the estimated brain age is often referred to as the “brain age gap” (Baecker et al., 2021) or “brain age delta” (Smith et al., 2019), as an approximation for accelerated or delayed aging of the brain. Correlation of the “brain age gap” with clinical factors indicated systolic/diastolic blood pressure, smoking habits, and cardiac function as predictors for accelerated aging (Cole, 2020). In contrast, higher bone mineral density was shown to be associated with delayed brain aging (Smith et al., 2019).

A comparison of different MRI approaches showed that diffusion tensor imaging (DTI) reached the best single modality performance in brain age estimation (*R*^2^ = 0.53, MAE = 3.9 years) which was similar to a multimodal approach using six MRI modalities (*R*^2^ = 0.62, MAE = 3.5 years) (Cole, 2020). Such multimodal models are based on a wide range of features, resulting in the need for a very high number of imaging data for good performance. In addition, the acquisition of multiparametric MRI is time consuming and thus limiting the usability in clinical routine. In comparison, DTI is acquired fast and provides comparable results across different scanners and field strengths, as demonstrated in a large-scale multicenter study with pooled data (Müller et al., 2016). This may become important in terms of a general application of the model since many brain age algorithms are sensitive to field strength or scanner type (Cole et al., 2017; Baecker et al., 2021).

In addition to applying ML to DTI data to assess aging (Cole, 2020), ML models based on DTI data have been utilized in clinical settings especially in neurodegenerative diseases (Dyrba et al., 2013; Sarica et al., 2017) or in order to stratify patient subgroups (Behler et al., 2022; Münch et al., 2022). Therefore, DTI data sets of patients and healthy participants are usually preprocessed to retrieve relevant anatomical / morphological information. For instance, such features can be extracted in a tract-based approach (Kocar et al., 2021b; Münch et al., 2022). As an alternative to clinical considerations, feature selection can also be done using statistical techniques (Talai et al., 2021). Neural networks were shown to be very performant in this context and in multiparametric MRI approaches, as well as support vector machines (Castellazzi et al., 2020; Kocar et al., 2021b; Tsai et al., 2022).

Quantification of physiological brain aging also plays a role in the analysis of clinical DTI studies. The regional differences in age-related changes in diffusion metrics and higher-order age dependencies, i.e., non-linear changes (Hsu et al., 2008; Westlye et al., 2010), result in the need to perform an optimized tract-based age correction (Behler et al., 2021). Given the limits of multimodal MRI-based brain age models, the questions arise: (1) whether a model that captures the interactions between white matter pathways might be just as accurate in the brain age estimation based on diffusion metrics and (2) whether such a model is also applicable to perform a tract-based age correction of DTI data in an automated approach. Since artificial neural networks (ANN) are powerful ML models, capable of finding patterns and interactions within data (Bishop, 2006), they are commonly used for brain age prediction. In contrast, age correction is almost exclusively done by simple linear regression, presumably because of the simplicity in generating its inverse function. This is impossible in non-linear multivariate regression models such as ANN; therefore, an algorithmic improvement is needed.

To this end, we fitted a multilayer perceptron (MLP) regression model as a type of ANN to a training set of tract-based diffusion metrics gathered from healthy adults. We examined the predictive performance of this model, compared the results to a ridge regression model, and conducted a thorough model inspection. Finally, we investigated how the ANN could be used for a tract-based non-linear age correction of diffusion metrics.

## Materials and methods

### Data collection and processing

The data set consisted of 219 healthy adults (103 male/116 female, mean age 51.6 ± 15.9 years, range 19.5–81.9 years, no diagnosed diseases) who underwent brain DTI and was previously used in a study by Behler and colleagues (Behler et al., 2021). The data were collected using three different scanners (1.5 T and 3 T field strength) with four different protocols, as summarized in Table 1. All subjects gave written informed consent. The present study was in accordance with institutional guidelines and approved by the Ethics Committee of Ulm University, Germany (reference # 19/12 and 279/19).

**TABLE 1:** Study population and imaging data.

Protocol	Age/years*	Number of subjects (m/f)	Field strength/T	Number of GD + (*b* = 0)	*b*-value/ (s/mm^2^)	Matrix (in-plane/slices)	Resolution/ mm^3^	TR/ms/ TE/ms
A	49.7 (29.3–62.6) 19.5–73.3	33 (17/16)	1.5	48 + 4	1,000	128 ×128/64	2.0 × 2.0 × 2.8	8,000/95
B	64.6 (60.2–69.4) 32.7–81.9	54 (23/31)	1.5	60 + 2	1,000	128 × 128/64	2.5 × 2.5 × 2.5	8,700/102
C	50.4 (44.7–57.0) 22.4–75.7	64 (34/30)	3.0	47 +1	1,000	96 × 128/52	2.2 × 2.2 × 2.2	7,600/85
D	46.5 (26.7–58.3) 20.9–79.5	68 (29/39)	3.0	64 + 1	1,000	128 × 128/81	1.7 × 1.7 × 1.7	7,600/85

*Given in median, (interquartile range), minimum—maximum. GD, gradient directions; TR, repetition time; TE, echo time.

For data preprocessing, the data were assessed for completeness and - according to an established analysis quality control (Müller et al., 2014)—corrupted gradient directions (GD) as well as motion artifacts were excluded from further analysis prior to correction of eddy current-induced geometric distortions. Following a standardized iterative stereotaxic normalization process, using scanning protocol-specific DTI template sets, data were transformed to the Montreal Neurological Institute (MNI) stereotaxic standard space. Maps of FA were calculated from MNI-normalized DTI data, and a Gaussian smoothing filter of 8 mm full width at half maximum (FWHM) was applied to the normalized individual FA maps. In order to calculate differences between scanning protocols at the group level, FA maps were harmonized according to an established harmonization procedure (Rosskopf et al., 2015; Müller et al., 2016). The FA maps of age-matched subsets of participants were arithmetically averaged separately for each scanning protocol. The resulting averaged FA maps were then used to calculate voxel-wise difference maps between protocol D and any other protocol since most participants underwent protocol D. The averaged three-dimensional (3-D) difference matrices, i.e., linear correction matrices, were then applied accordingly to the FA maps of all participants who underwent scanning protocols A, B, or C. This procedure resulted in the recalibration of all FA maps acquired with different protocols and the harmonization of subject groups.

For the analysis of fiber tract (FT), specific tracts were identified by using a seed-based approach based on an averaged DTI dataset. The modified deterministic streamline tracking approach (Mori et al., 2002; Müller et al., 2009) used an eigenvector scalar product threshold of 0.9 and considered only voxels with an FA value above a threshold of 0.2 [cortical gray matter shows FA values up to 0.2 (Kunimatsu et al., 2004)]. Regions of interest (ROIs) with a radius of between 6 and 10 mm were defined for the seed regions. All FTs originating in the seed ROI or multiple ROIs for extended seed regions (e.g., callosal areas), respectively, define the corresponding tracts of interest (TOIs). The following 21 TOI were identified using this seed-based fiber tracking approach: Corticospinal tract (CST), frontooccipital tract, fasciculus uncinatus, optic radiation, superior longitudinal fasciculus (SLF), inferior longitudinal fasciculus (ILF), cingulum, superior cerebellar peduncle (SCP), middle cerebellar peduncle (MCP), corticostriatal tract, corticopontine tract, corticorubral tract, perforant path, the tract from temporal lobe to hypothalamus, anterior limb of the inner capsule, posterior limb of the inner capsule, and the tracts associated with the corpus callosum areas I to V. FA values were calculated by arithmetic averaging of the bihemispheric data.

### Machine learning model construction

The orientational dependence of the voxel-wise diffusion tensor can be performed in several combinations of the Eigenvectors, resulting in the scalar parameters fractional anisotropy (FA), mean diffusivity, axial diffusivity, and radial diffusivity, which each have advantages in specific research contexts. In order to avoid redundancy, we selected FA for the analyses performed in the current study, which already showed to be a robust representation of (age-related) diffusion properties (Salat et al., 2005; Behler et al., 2021; Kocar et al., 2021a; Münch et al., 2022). The selection of the TOI was performed in such a way that various functional areas and diffusion directions were covered. Clinical significance of these tracts has been shown, both for aging (Behler et al., 2021) and neurodegenerative diseases (Kocar et al., 2021b). Beyond this preprocessing, no further restrictions preceded the construction of our models. Although incomplete samples were not a problem in the original data set regression analysis, here, they represent one in the application of more complex ML models. Therefore, two samples had to be discarded from the initial data set due to a missing FA value in one tract. From the remaining 217 samples, a training and a test data set were defined by a random 80:20 split. Within the training data set, leave-one-out cross validation (LOOCV) was used for hyperparameter tuning (Hastie et al., 2017). All FA values were z-transformed based on the training data set for calibration. Rescaling of the target variable (age) was performed to reduce the computational load during ML model calculation. Conversely, for data presentation, the transformation was reversed.

Using the scikit-learn 0.24.2 library for python (Pedregosa et al., 2011), two ML models were implemented, a ridge regression and by an introduction of a hidden layer (as an extension to the ridge regression) an MLP regression. Ridge regression is a robust model for statistical regression even in the presence of collinearity (Leeuwenberg et al., 2022). MLP as a type of ANN is highly efficient in finding complex interactions within data and are overall powerful algorithms, given enough data and a sound construction process (Bishop, 2006). Unless stated otherwise, the default hyperparameters of the Ridge classes were used: fit_intercept = True, max_iter = None, tol = 0.001, solver = “auto,” positive = False, random_state = None. The same applies to the MLP Regressor: solver = “adam,” batch_size = “auto,” learning_rate_init = 0.001, shuffle = True, random_state = None, tol = 0.0001, warm_start = False, early_stopping = False, beta_1 = 0.9, beta_2 = 0.999, epsilon = 1e-08, n_iter_no_change = 10. In the ridge regression model, the L2-regularization parameter was determined by an exhaustive grid-search, ranging from 100 to 0 with a decrement of 0.1. Each value in the grid was tested by LOOCV and the best value in terms of the least squared error as a performance metric was chosen for the final model, which was trained on the entire training data set.

**FIGURE 1:**
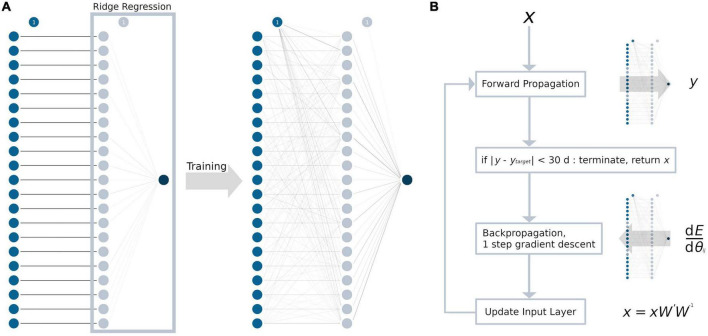
Development of the ANN and the ANN age-correction algorithm. Circles represent neurons, the bias unit is marked with “1.” For every tract system (input layer, blue), there is exactly one neuron in the hidden layer (light blue). Weights are displayed as lines in grayscale, with 0 as white and 1 as black. **(A)** The weights between the input and the hidden layer were initialized as the identity matrix. The weights between hidden layer and the output layer (dark blue) were initialized as the coefficients from the ridge regression (light blue box). After training, the hidden layer processes multiple interactions from the input layer, which are represented by gray lines. **(B)** ANN age correction algorithm: First, the input data *x* were forward propagated through the ANN with the weight matrix *W*, resulting in the brain age prediction *y*. Then, the difference between *y* and the age correction target *y*_target_ (= error *E*) was backpropagated to calculate the modified weight matrix *W’*. Forward and backpropagation are identical to a regular gradient descent algorithm which are commonly used for ANN training. Last, the input data *x* were updated by matrix multiplication (*xW’W^– 1^*). These steps were repeated until the error *E* reached an absolute value of 1 month or less and the algorithm terminated. d*E*/d*θ*_*ij*_ = partial derivatives of the error with respect to the weights in the weight matrix *W*.

The MLP contained only one hidden layer with a single weight matrix, given the amount of data and overall model complexity. For every TOI in the input layer, we chose to have one neuron in the hidden layer, resulting in 21 neurons and a square weight matrix. A graphical abstraction of the model building process is presented in Figure 1A. As an activation function, a rectified linear unit (ReLU) (Glorot et al., 2011) was used, as it resembles the complex relationship between age and tract-based FA values. During adulthood, an age range without changes in the FA values is followed by a linear decrease (Westlye et al., 2010; Behler et al., 2021). In addition to L2-regularization, non-random initialization and pruning were used to prevent overfitting (Kukaèka et al., 2017). For initializing the weight matrix, an identity matrix was used (Le et al., 2015). This not only aimed to prevent overfitting but also increased the explicability of the model, as the neurons in the hidden layer may retain some resemblance to their respective input neuron after training. All biases between the input layer and the hidden layer were set to 0. Between the hidden layer and the output layer, the coefficients from the trained ridge regression model for weight initialization and the intercept for bias initialization were used as a pre-trained last layer. Training was conducted by first forward propagating the training sample’s data through the initialized model and then backpropagating the loss function (= squared error ∧ L2-regularization) through the hidden layers by calculating the partial derivatives with respect to the model parameters. Then, the weights and biases were adjusted using the gradient descent algorithm. This procedure was repeated for up to a maximum of 100,000 iterations to ensure convergence. After training the entire model, the weights of the neural network were pruned by setting the lowest absolute value to 0, until there was at least one sample for each parameter (Han et al., 2015; Blalock et al., 2020). Similar to the ridge regression model, the L2-regularization parameter was determined by an exhaustive grid search, ranging from 20 to 0 with a decrement of 0.1 and choosing the best value by LOOCV.

### Machine learning model inspection

The coefficients of the ridge regression model were analyzed as a proxy for feature importance. For the ANN, the permutation importance for each feature was calculated using the test data set and 1,000 iterations (Breiman, 2001). For further insights, the weights of the ANN were inspected. Weights converging on a single hidden layer neuron were taken as an indication of possible interactions between the respective input TOIs.

### Brain age prediction

Brain age prediction was conducted on the validation and test data set using both ridge regression and the ANN. For evaluating the performance of the models, the *R*^2^ value and the mean absolute error (MAE) were calculated. The 95%-confidence intervals (CI) were determined by bootstrapping with 1,000 iterations (Efron and Tibshirani, 1993). In addition, the distributions of the prediction error, i.e., the difference between chronological age and predicted brain age, were tested for normality using the Shapiro-Wilk test (Shapiro and Wilk, 1965).

### Age correction

Age correction of the tract-based FA values was conducted for samples in the test data set with the test samples’ mean chronological age as the global target age. The basic structure of the age correction approach outlined here is adopted from an image generation algorithm that combines the artistic style from one image and the content from another image (Gatys et al., 2015, 2017). In summary, the gradient descent algorithm was applied not to update the weights and biases of the ANN, but to modify the input data (Figure 1B). First, the tract-based FA values (= input data *x*) from each sample were forward propagated through the ANN, putting out the predicted brain age *y*. Then, the age difference between the sample’s chronological age and the global target age was added to the predicted brain age *y* to define the sample’s age correction target *y*_*target*_. Note, that *y*_*target*_ cannot be set to the global target age, as this would lead not only to the desired age correction, but also the correction of the “brain age gap.” To modify the input data *x*, the difference between *y* and *y*_*target*_ (= error *E*) was backpropagated through the ANN. The hyperparameters of the ANN were set to warm_start = True, max_iter = 1, solver = “sgd” and alpha = 0, and exactly one step of gradient descent was conducted to calculate the modified weight matrix *W’*. Finally, instead of updating the weights and biases of the model like in a regular gradient descent algorithm, the input data were updated. The updated input data were calculated as the dot-product of the input data *x*, the modified weight matrix *W’*, and the inverse of the original weight matrix *W*. Adjustment of the input data was restricted by the following rules:

1. If the global target age is higher than the chronological age, FA values of the individual sample must decrease.
2. If the global target age is lower than the chronological age, FA values of the individual sample must increase.
3. The FA values derived from the superior and MCP (SCP and MCP) should remain unchanged, as these tracts generally do not exhibit age-associated alterations (Cox et al., 2016; Behler et al., 2021).

For any value that did not meet these requirements, the initial value was retained. Forward propagation, backpropagation and updating the input data were repeated until the error *E* reached an absolute value of less than 1 month. Then, the algorithm was terminated and the modified input data were considered age-corrected. When processing these new age-corrected input data with the ANN, the output age differs from the original predicted age by the difference between the sample’s chronological and the global target age. The algorithm corrects all tract-based FA values to the same target age and cannot introduce tract-specific age targets, as in a tract-wise age correction.

The ANN-based age-corrected FA values were compared with those that were age-corrected using a tract-specific linear regression approach proposed by Behler and colleagues (Behler et al., 2021). As a performance metric, the *R*^2^ values and 95% confidence intervals were calculated by bootstrapping with 1,000 iterations, both for the individual tracts and the combined data set. In addition, Bland-Altman analysis was performed for the individual tracts and the combined data set (Bland and Altman, 1986).

## Results

### Model construction and inspection

**FIGURE 2:**
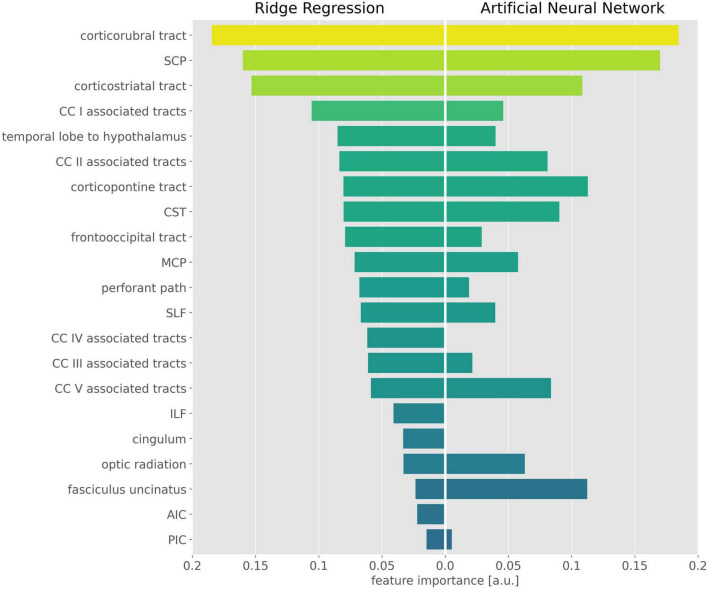
Important features in the ridge regression and the artificial neural network (ANN). Feature importance is displayed as the absolute value of the ridge regression coefficients **(Left)** and the mean permutation importance (PI) values from the ANN **(Right)**. For better visualization, PI values were rescaled and negative PI values were set to 0. CST, corticospinal tract; SLF, superior longitudinal fasciculus; ILF, inferior longitudinal fasciculus; SCP, superior cerebellar peduncle; MCP, middle cerebellar peduncle; AIC, anterior limb of internal capsule; PIC, posterior limb of internal capsule; CC, corpus callosum.

The exhaustive grid searches determined the L2-regularization term as alpha = 62.4 for the ridge regression and alpha = 10.5 for the ANN. Pruning of the ANN resulted in a sparse weight matrix with 67% zeroes. The coefficients of the ridge regression are reported in Table 2, the intercept was 0. Parameter inspection of the ANN showed that the main diagonal of the weight matrix contained the highest value for each hidden layer neuron (Figure 1 and Supplementary Table 1), retaining the initial relationship to their input TOI. In the hidden layer neurons 4, 8, 14, and 21, respectively, 10 or more non-zero weights from the input layer converged. With respect to their largest weight, these hidden layer neurons corresponded to the optic radiation, SCP, temporal lobe to hypothalamus, and corpus callosum area V-associated tracts. Permutation importance indicated that features with large coefficients in the ridge regression were also important for brain age prediction in the ANN, most notably the corticorubral tract, the SCP, and the corticostriatal tract (Figure 2).

**TABLE 2:** Ridge regression coefficients.

Tract of interest	Ridge regression coefficient
CST	–0.08
Frontooccipital tract	–0.08
Fasciculus uncinatus	0.02
Optic radiation	–0.03
SLF	0.07
ILF	–0.04
Cingulum	–0.03
SCP	0.16
MCP	–0.07
Corticostriatal tract	–0.15
Corticopontine tract	0.08
Corticorubral tract	–0.18
Perforant path	–0.07
Temporal lobe to hypothalamus	0.09
AIC	–0.02
PIC	–0.01
CC I associated tracts	–0.11
CC II associated tracts	–0.08
CC III associated tracts	–0.06
CC IV associated tracts	–0.06
CC V associated tracts	0.06

CST, corticospinal tract; SLF, superior longitudinal fasciculus; ILF, inferior longitudinal fasciculus; SCP, superior cerebellar peduncle; MCP, middle cerebellar peduncle; AIC, anterior limb of internal capsule; PIC, posterior limb of internal capsule; CC, Corpus Callosum.

### Age prediction

The following results are reported as the mean, with the 95% CI in square brackets. Ridge regression achieved an *R*^2^ value of 0.38 [0.27; 0.48] in the LOOCV and 0.38 [0.10; 0.59] in the test data set. The MAE was 10.59 [9.53; 11.76] years in the LOOCV and 8.36 [6.71; 10.29] years in the test data set. The ANN achieved an *R*^2^ value of 0.47 [0.36; 0.56] in the LOOCV and 0.47 [0.23; 0.66] in the test data set. The MAE was 9.93 [8.95; 10.98] years in the LOOCV and 7.61 [6.01; 9.53] years in the test data set. Prediction errors in the ANN and in the ridge regression were normally distributed with *p* > 0.05 (also see Supplementary Figure 1).

### Age correction

**FIGURE 3:**
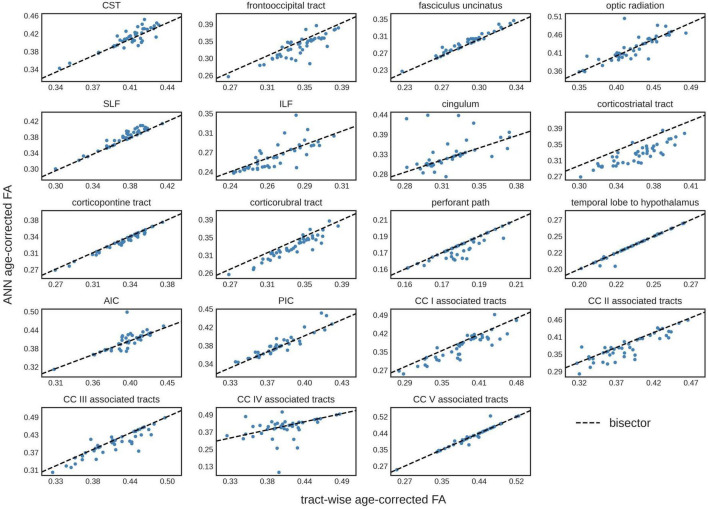
Tract-wise comparison of two different approaches for age correction of fractional anisotropy (FA) values. The tract-wise linear age-corrected FA values is plotted against the artificial neural network (ANN) age-corrected FA values of each subject in the test data set (*N* = 43). The bisector is displayed as a “guide for the eye.” CST, corticospinal tract; SLF, superior longitudinal fasciculus; ILF, inferior longitudinal fasciculus; AIC, anterior limb of internal capsule; PIC, posterior limb of internal capsule; CC, corpus callosum.

The comparison of the ANN-based age-corrected FA with the tract-wise linear age-corrected FA resulted in an *R*^2^ value of 0.90 [0.82; 0.93] and a mean difference of 0.00 [-0.04; 0.05] for all tract systems combined. The tract-wise comparison of both approaches showed an inconsistent pattern, with the frontal tracts showing more concordance than the posterior ones. Low or negative *R*^2^ values were noted in the frontooccipital tract, the optic radiation, the ILF, the cingulum, and the tracts associated with segment IV of the corpus callosum. The Bland-Altman analysis indicated that FA values were systematically lower after ANN vs. tract-wise linear age correction in the corticostriatal tract. For a complete overview, see Table 3 and Figure 3.

**TABLE 3:** Performance metrics of the ANN-based age correction of fractional anisotropy (FA) compared to a tract-wise linear age-correction.

Tract of interest	*R*^2^ [95% CI]	Mean difference [limits of agreement]
CST	0.68 [0.28; 0.88]	0.00 [−0.02; 0.02]
Frontooccipital tract	0.34 [−0.41; 0.72]	0.01 [−0.02; 0.04]
Fasciculus uncinatus	0.92 [0.85; 0.96]	0.00 [−0.01; 0.00]
Optic radiation	0.57 [−0.54; 0.85]	0.00 [−0.04; 0.04]
SLF	0.84 [0.67; 0.94]	−0.01 [−0.02; 0.01]
ILF	0.20 [−0.97; 0.63]	0.00 [−0.03; 0.03]
Cingulum	−1.85 [−6.39; 0.11]	−0.01 [−0.08; 0.06]
SCP	1.00 [1.00; 1.00]	0.00 [0.00; 0.00]
MCP	1.00 [1.00; 1.00]	0.00 [0.00; 0.00]
Corticostriatal tract	−0.60 [−1.45; −0.01]	0.03 [0.00; 0.05]
Corticopontine tract	0.96 [0.92; 0.98]	0.00 [0.00; 0.01]
Corticorubral tract	0.57 [0.31; 0.77]	0.01 [−0.01; 0.03]
Perforant path	0.68 [0.33; 0.86]	0.00 [−0.01; 0.01]
Temporal lobe to hypothalamus	0.96 [0.81; 1.00]	0.00 [−0.01; 0.01]
AIC	0.35 [−1.87; 0.85]	0.00 [−0.04; 0.03]
PIC	0.80 [0.62; 0.90]	0.00 [−0.02; 0.01]
CC I associated tracts	0.62 [0.34; 0.79]	0.02 [−0.03; 0.06]
CC II associated tracts	0.61 [0.30; 0.79]	0.01 [−0.03; 0.05]
CC III associated tracts	0.72 [0.40; 0.86]	0.01 [−0.02; 0.05]
CC IV associated tracts	−2.66 [−11.99; −0.20]	0.01 [−0.12; 0.14]
CC V associated tracts	0.95 [0.76; 0.99]	0.00 [−0.02; 0.02]

*R*^2^ values are displayed with the 95% confidence interval (CI). The mean differences between both age-corrected FA-values and the limits of agreement were obtained by Bland-Altman analyses.

## Discussion

### Artificial neural network for age correction based on diffusion metrics

In the present study, we provided the proof of concept for non-linear age correction of tract-based diffusion metrics data using an ANN. First, the ANN was trained on tract-based DTI metrics gathered from healthy adults, then, the ANN was applied to a separate test data set for age correction, using a modified gradient descent algorithm. The comparison with a state-of-the-art linear tract-wise age-correction (Behler et al., 2021) gave an *R*^2^ value of 0.90. As an overall pattern, ANN age correction was more concordant with tract-wise age correction in frontal areas, less in occipital or infratentorial areas.

### Artificial neural network explained about 50% of the subjects’ age

In the current approach, the ANN explained about 50% of the subjects’ chronological age by white matter diffusion metrics alone, which is in line with previously reported performance metrics (Cole, 2020; Niu et al., 2020).

In brain age prediction models, the difference between the predicted brain age and the chronological age is often referred to as the “brain age gap” and is considered an indicator for accelerated or delayed brain aging. The age prediction errors of the ANN were normally distributed. Biological markers and their measuring errors generally follow a normal distribution which is often explained by central field theory (Lyon, 2014). We regard the normal distribution of the age prediction errors of the ANN as consistent with the idea that both brain age and the “brain age gap” can be regarded as biomarkers (Franke and Gaser, 2019).

Some lifestyle and biographical factors such as alcohol consumption (McEvoy et al., 2018), body composition (Beck et al., 2022), or previous childbirths (Voldsbekk et al., 2021) are associated with white matter alterations. If a detailed description of these factors is available in a given study population, this information could be included in the model and corrections for factors other than age could be possible. Given the difficulties in obtaining these data in retrospective clinical studies, we suggest applying the proposed ANN age-correction at the group level only, where the influence of different biographies and lifestyles should be averaged out. In addition to physiological aging, diseases such as alcohol dependence can have a long-term impact on DTI metrics (Pfefferbaum et al., 2014). As the ANN was trained on data of healthy participants with no known pathological conditions or cognitive deficits, it cannot take disease-specific alterations into account. In patient data, disease-specific tract data would not be modified beyond what can be explained by physiological aging alone. In turn, disease-age interactions cannot be accounted for.

### Tract-specific predictive contributions

Model inspection revealed high importance of the corticorubral tract, the SCP, and the corticostriatal tract in brain age prediction and indicated interaction effects in optic radiation, SCP, the tract from temporal lobe to hypothalamus, and corpus callosum area V-associated tracts. The importance of the SCP was surprising, as previous studies have shown no age-related changes in diffusion metrics in cerebellar tracts (Behler et al., 2021). This result could be explained by the SCP serving as a reference against which the frontal areas were compared. Accordingly, non-specific changes in whole-brain FA (e.g., due to lifestyle) would not automatically change the age prediction, since the SCP would partially level out the changes in the frontal lobe. If this interpretation is correct, the proposed ANN may even account for some of the lifestyle factors, even if they are not explicitly presented to the model.

### Non-linear age correction can be performed by artificial neural network

The comparison of ANN age-corrected FA values with age-corrected FA based on an established tract-wise method showed similar results with a good overall performance (*R*^2^ = 0.90). Nevertheless, the ANN struggled to accurately correct tracts in posterior brain areas, namely the frontooccipital tract, the optic radiation, the ILF, the cingulum, and the tracts associated with area IV of the corpus callosum. Inaccuracies during ANN age-correction compound with every iteration of gradient descent and there is no mechanism to limit the range of possible values. For training ML models, this limitation is generally implemented by L2-regularization. The lack of such a feature in our ANN age correction algorithm might explain the more pronounced deviations found in the cingulum and the corpus callosum area IV associated tracts (see Figure 3), where implausibly high/low FA-values were suggested as age-corrected by the ANN. The feed-forward nature of the age-correction algorithm (see Figure 1B) may provide an explanation for some of the outliers. In theory, the issue could be addressed by introducing L2-regularization to the outlined method of ANN age correction. However, the implementation might prove challenging, as high L2-parameters would prioritize FA-values close to 0 (z-transformed mean) over reducing the actual error term. A more practical approach could be to use ANN age-correction only when the amount of intended age-correction is low.

ANN-based non-linear age correction generates synthetic data for the intended target age. These synthetic tract-based diffusion data could not only be used for age correction in group studies but could also be for data augmentation and sharing. Data augmentation, which can also be considered a form of regularization (Kukaèka et al., 2017), is common in advanced ML techniques such as deep neural networks (Abadi et al., 2016). In various neurodegenerative diseases, ML models based on neuroimaging data can strengthen diagnostic accuracy (Lampe et al., 2022). However, collecting a large amount of neuroimaging data in rare brain diseases is often challenging, as it is to train complex yet accurate ML models (Castiglioni et al., 2021). The data generation used here for age correction might be used for data augmentation of limited diffusion metric data sets by using the existing data sets to create new ones.

### Limitations

The present study is not without limitations. The relatively small sample size (*n* = 217) prompted us to prune the weight matrix to reduce model complexity. Given this sample size, we also decided against performing subgroup analyses, e.g., of gender differences. While we assumed that our subjects were healthy based on the absence of diseases, the finding of accelerated brain aging in some subjects may contradict this notion. Possible underlying factors were not investigated in the present study. There were no signs of impaired neurological and neurocognitive functioning among the study participants according to medical history and clinical impression, however, no standardized neurocognitive screening was performed.

DTI measures a physical parameter, i.e., the results should theoretically be the same for every protocol independent of the scanner. However, there are differences in DTI parameters occurring from different values for TE, B0, and voxel volumes. In order to reduce these effects, a (linear) harmonization of FA values from different protocols has been applied. The differences measured in FA maps of age-matched (healthy) controls can be used for this task (Müller et al., 2016). The use of age-matched control groups enabled us to calculate the protocol-based differences, at the expense of small differences of age-matched subject groups that are assumed to be one or two orders of magnitude lower than protocol differences.

## Conclusion

In conclusion, the present study provided a proof of concept for the use of ANN in non-linear age correction of tract-based diffusion metrics. Future studies could extend the proposed method of ANN age correction to data augmentation.

## Data availability statement

The data that support the findings of this study are available from the corresponding author, TK, upon reasonable request. Requests to access these datasets should be directed to thomas.kocar@uni-ulm.de.

## Ethics statement

The studies involving human participants were reviewed and approved by the Ethics Committee of Ulm University, Germany (reference # 19/12 and 279/19). The patients/participants provided their written informed consent to participate in this study.

## Author contributions

TK: study concept and design, analyses and interpretation of data, and drafting of manuscript. AB and JK: study concept and design, interpretation of data, and critical revision of manuscript for intellectual content. CL, MD, and AL: interpretation of data and critical revision of manuscript for intellectual content. H-PM: study concept and design, analyses and interpretation of data, and critical revision of manuscript for intellectual content. All authors contributed to the article and approved the submitted version.
