# Supplementary material for: Artificial neural networks for non-linear age correction of diffusion metrics in the brain
Source: Front Aging Neurosci. 2022 Oct 20;14:999787. doi: 10.3389/fnagi.2022.999787 (PMC9632350; doi:10.3389/fnagi.2022.999787)
Supplement: Supplementary file 1 [file Data_Sheet_1.docx]

Supplementary Material

# Supplementary Figures and Tables

## Supplementary Tables

**Supplementary Table 1: ANN weight matrix**. N1, N2, … N21 represent the neurons in the hidden layer. I1, I2, … I21 represent the neurons in the input layer. The weights are denoted as a matrix / 2-dimensional array. The largest weight for each hidden layer neuron is highlighted.

|  |  | **N1** | **N2** | **N3** | **N4** | **N5** | **N6** | **N7** | **N8** | **N9** | **N10** | **N11** | **N12** | **N13** | **N14** | **N15** | **N16** | **N17** | **N18** | **N19** | **N20** | **N21** |  |
| --- | --- | --- | --- | --- | --- | --- | --- | --- | --- | --- | --- | --- | --- | --- | --- | --- | --- | --- | --- | --- | --- | --- | --- |
| **I1** | [[ | 0.26, | 0, | 0, | 0.16, | 0, | 0, | 0, | 0.07, | 0, | 0, | 0, | 0.09, | 0, | 0.09, | 0, | 0, | 0.05, | 0, | 0, | 0.05, | 0.19 | ], |
| **I2** | [ | 0, | 0.24, | 0 | 0.12, | 0, | 0, | 0.04, | 0.15, | 0, | 0, | 0, | 0, | 0, | -0.09, | 0, | 0, | 0, | 0, | 0, | 0.05, | 0 | ], |
| **I3** | [ | 0, | 0, | 0.23, | 0, | 0, | 0, | 0, | 0.20, | 0, | 0, | 0, | 0.05, | 0, | 0.09, | 0, | 0, | 0, | 0, | 0, | -0.05, | 0 | ], |
| **I4** | [ | 0, | 0, | 0, | 0.29, | 0, | 0, | -0.05, | 0, | 0, | -0.05, | 0, | 0, | 0, | 0, | 0, | 0, | 0, | 0, | 0, | 0.06, | 0.10 | ], |
| **I5** | [ | 0, | 0, | 0, | -0.09, | 0.22, | 0, | 0, | 0.05, | 0.05, | 0, | 0, | 0, | 0, | 0, | 0, | 0, | 0, | 0, | 0, | 0, | 0 | ], |
| **I6** | [ | 0, | 0.05, | 0, | 0, | 0, | 0.24, | 0, | 0, | 0, | 0, | 0, | 0, | 0, | 0, | 0, | 0, | 0, | 0, | 0, | -0.05, | 0.10 | ], |
| **I7** | [ | 0, | 0.04, | 0, | 0.13, | 0, | 0, | 0.26, | -0.06, | 0, | 0, | 0, | 0, | 0, | 0, | 0, | 0.10, | 0, | 0, | 0, | 0, | -0.06 | ], |
| **I8** | [ | 0.09, | 0, | 0, | -0.09, | 0, | 0, | 0, | 0.30, | 0.06, | 0, | 0, | -0.08, | 0, | 0, | 0, | 0, | -0.06, | -0.05, | 0, | 0.08, | 0 | ], |
| **I9** | [ | 0.05, | 0, | 0, | 0.14, | 0, | 0, | 0.13, | 0, | 0.27, | 0, | 0, | 0.07, | 0, | 0, | 0, | 0, | 0.16, | 0, | 0, | 0, | -0.08 | ], |
| **I10** | [ | 0, | 0.12, | 0, | 0, | 0, | 0.06, | 0.08, | -0.08, | 0, | 0.26, | 0, | 0, | 0, | -0.09, | 0.05, | 0, | 0, | 0, | 0, | 0, | -0.04 | ], |
| **I11** | [ | 0, | 0, | 0, | 0.10, | 0, | 0, | -0.04, | 0, | 0.04, | 0, | 0.21, | 0, | 0, | 0.07, | 0, | 0, | -0.09, | -0.07, | 0, | 0, | 0 | ], |
| **I12** | [ | 0.11, | 0.09, | 0, | 0.09, | 0, | 0, | 0, | 0.15, | -0.08, | 0, | 0, | 0.28, | 0, | 0.18, | 0.05, | 0.08, | 0, | 0, | 0, | 0, | 0.04 | ], |
| **I13** | [ | 0, | 0.06, | 0, | 0, | 0, | 0, | 0, | 0.18, | 0, | 0, | 0.05, | 0, | 0.22, | -0.06, | 0, | 0, | 0, | 0, | 0, | 0, | -0.08 | ], |
| **I14** | [ | -0.06, | 0, | 0, | 0.11, | 0, | 0, | 0.04, | 0.06, | 0, | 0, | 0, | 0, | 0, | 0.26, | 0, | 0, | -0.09, | 0, | 0, | 0, | -0.08 | ], |
| **I15** | [ | 0, | 0.06, | 0, | 0, | 0, | 0, | -0.07, | 0.10, | 0, | 0, | 0, | 0.05, | 0, | 0, | 0.23, | 0, | 0, | -0.05, | 0, | 0, | 0 | ], |
| **I16** | [ | 0.06, | 0, | 0, | -0.04, | 0, | 0, | 0, | 0, | 0, | 0, | 0, | 0.11, | 0, | 0, | 0, | 0.25, | -0.09, | 0, | 0, | 0, | 0 | ], |
| **I17** | [ | 0, | 0, | 0, | 0, | 0, | 0, | 0, | 0.12, | 0, | 0, | 0, | 0, | 0, | 0, | 0, | 0, | 0.27, | 0.05, | 0, | 0, | -0.05 | ], |
| **I18** | [ | 0, | 0, | 0, | 0.14, | 0, | 0, | 0, | 0.10, | 0, | 0, | 0, | 0, | 0, | 0, | 0, | 0, | 0.09, | 0.23, | 0, | 0, | 0 | ], |
| **I19** | [ | 0.05, | 0, | 0, | 0, | 0, | 0, | 0, | 0.12, | 0, | 0, | 0, | 0, | 0, | 0.12, | 0, | 0.04, | 0, | 0, | 0.25, | 0.06, | 0.09 | ], |
| **I20** | [ | 0.05, | 0, | 0, | 0, | 0, | 0, | 0, | 0.10, | 0, | 0, | -0.04, | 0.05, | 0, | -0.08, | 0, | 0, | 0, | 0, | 0.06, | 0.25, | 0.08 | ], |
| **I21** | [ | 0, | 0, | 0, | 0.11, | 0, | 0, | 0, | 0, | 0, | 0, | -0.05, | 0, | 0, | 0.12, | 0, | 0, | -0.07, | -0.05, | 0.07, | 0.11, | 0.36 | ]] |

## Supplementary Figures
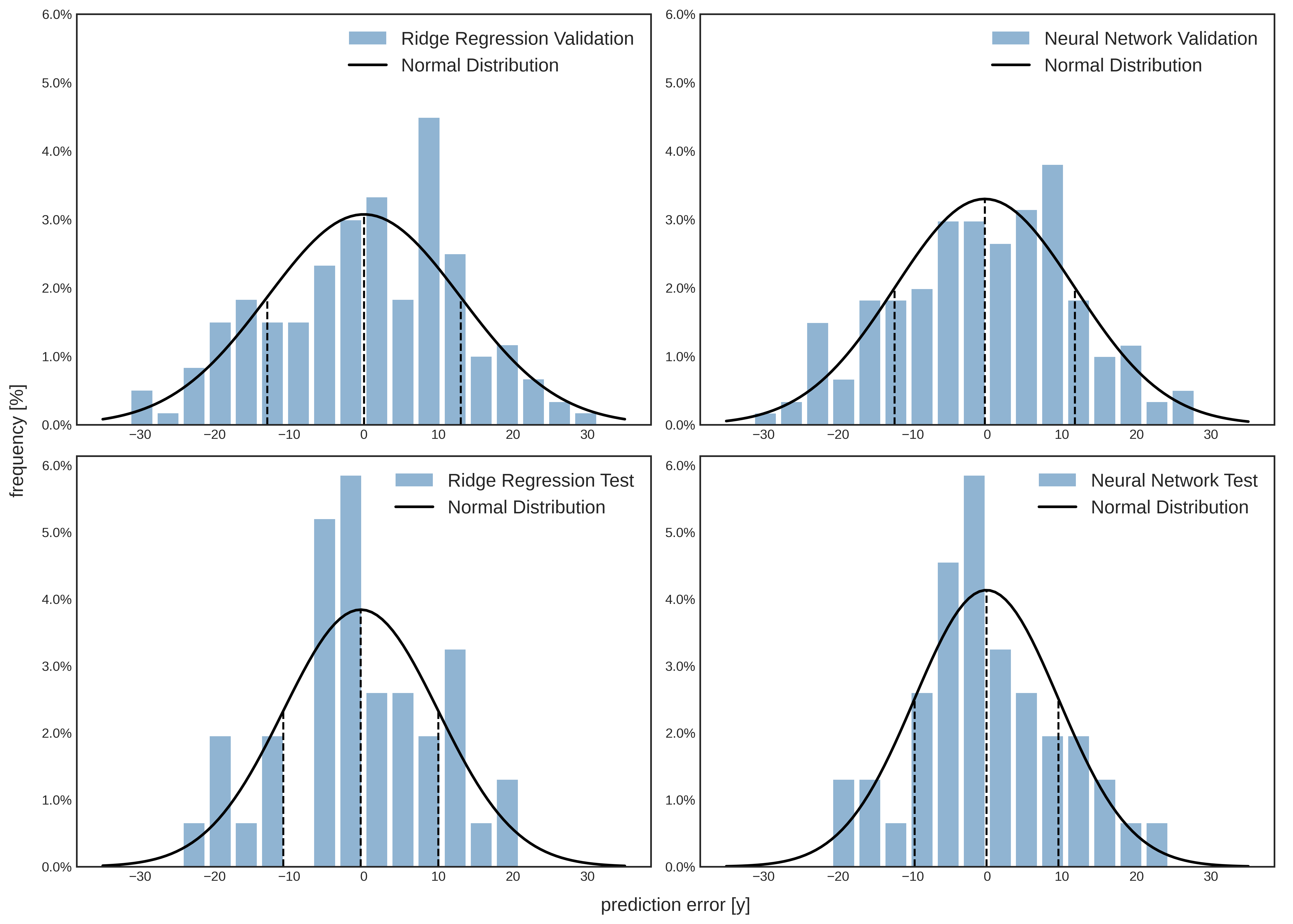


**Supplementary Figure 1:** Age prediction error distribution in years. Histogram of the LOOCV (validation) and the test data, using ridge regression (left) and MLP Regression / Neural Network (right). A normal distribution curve was calculated and overlayed. The mean and the standard deviation are marked with a dashed line.
